# Proteomics of *Trypanosoma evansi* Infection in Rodents

**DOI:** 10.1371/journal.pone.0009796

**Published:** 2010-03-22

**Authors:** Nainita Roy, Rishi Kumar Nageshan, Rani Pallavi, Harshini Chakravarthy, Syama Chandran, Rajender Kumar, Ashok Kumar Gupta, Raj Kumar Singh, Suresh Chandra Yadav, Utpal Tatu

**Affiliations:** 1 Department of Biochemistry, Indian Institute of Science, Bangalore, India; 2 National Research Centre on Equines, Hisar, India; Swiss Federal Institute of Technology Lausanne, Switzerland

## Abstract

**Background:**

*Trypanosoma evansi* infections, commonly called ‘surra’, cause significant economic losses to livestock industry. While this infection is mainly restricted to large animals such as camels, donkeys and equines, recent reports indicate their ability to infect humans. There are no World Animal Health Organization (WAHO) prescribed diagnostic tests or vaccines available against this disease and the available drugs show significant toxicity. There is an urgent need to develop improved methods of diagnosis and control measures for this disease. Unlike its related human parasites *T. brucei* and *T. cruzi* whose genomes have been fully sequenced *T. evansi* genome sequence remains unavailable and very little efforts are being made to develop improved methods of prevention, diagnosis and treatment. With a view to identify potential diagnostic markers and drug targets we have studied the clinical proteome of *T. evansi* infection using mass spectrometry (MS).

**Methodology/Principal Findings:**

Using shot-gun proteomic approach involving nano-lc Quadrupole Time Of Flight (QTOF) mass spectrometry we have identified over 160 proteins expressed by *T. evansi* in mice infected with camel isolate. Homology driven searches for protein identification from MS/MS data led to most of the matches arising from related Trypanosoma species. Proteins identified belonged to various functional categories including metabolic enzymes; DNA metabolism; transcription; translation as well as cell-cell communication and signal transduction. TCA cycle enzymes were strikingly missing, possibly suggesting their low abundances. The clinical proteome revealed the presence of known and potential drug targets such as oligopeptidases, kinases, cysteine proteases and more.

**Conclusions/Significance:**

Previous proteomic studies on Trypanosomal infections, including human parasites *T. brucei* and *T. cruzi*, have been carried out from lab grown cultures. For *T. evansi* infection this is indeed the first ever proteomic study reported thus far. In addition to providing a glimpse into the biology of this neglected disease, our study is the first step towards identification of diagnostic biomarkers, novel drug targets as well as potential vaccine candidates to fight against *T. evansi* infections.

## Introduction

*T. evansi* is a widely-distributed, monomorphic protozoan which infects large animals such as equines, camels, dogs, cattle and deer. Epidemics of the disease called ‘surra’ which is caused by *T. evansi* infections results in the death of thousands of animals amongst which horses are the most susceptible [1]. *T. evansi* is geographically distributed in Asia, Africa and South America. With the number and severity of surra outbreaks escalating radically over the last few years, this protozoan parasite has a significant impact on the increasing mortality rates of livestock across the world. *T. evansi* is mechanically transmitted by blood-sucking insect species such as *Tabanus*, *Stomoxys*, *Lypersoia* and *Haematopota*. Keeping in view the large economic losses caused by this disease, an ad hoc group of Organisation Internationale des Epizootie (O.I.E.) on Non Tsetse Transmitted Animal Trypanosomes (NTTAT) was set up under the sponsorship of Food and Agriculture Organization (F.A.O.), Division of Production and Animal Health and International Livestock Research Institute (I.L.R.I.) in relationship with World Health Organization (W.H.O.) during the O.I.E. general session by representatives of above organizations and O.I.E. delegates of Asia, Africa and Europe. Compared to its closely related species *T. brucei* and *T. cruzi*, which infect humans, less attention is being given to *T. evansi* infections. While *T. evansi* infections are mainly restricted to animals, a recent report suggested its ability to jump species barrier and infect humans, in a minority of population having defect in ApoL1 [2].

In contrast to the closely related species *T. brucei* [3], [4], *T. evansi* lacks the maxicircle kinetoplastid DNA. The loss of maxicircle kDNA in T. evansi has been shown to lock the *trypanosome* in the bloodstream stages in vertebrates where the parasite multiplies by longitudinal binary fission. The absence of intermittent development in any insect vector has enabled *T. evansi* to spread beyond the tse-tse fly belt of Africa to other areas such as Asia, central and South America [5].

There is no vaccine for prevention of surra. The lists of drugs that are commonly used for treatment include suramin, diminazene aceturate, quinapyramine and cymelarsan [6], [7]. While effective, toxic side effects are exhibited by many of these drugs and exposure to sub curative doses of trypanocides in field is known to give rise to drug resistance. Development of drug resistance in T. evansi was reported from India [8] and Sudan [9]. Therefore there is a need to find more effective and relatively harmless treatment strategies. Despite the growing incidence of this disease, there are relatively few prescribed diagnostic test for timely detection of this infection. Practiced diagnostic techniques include wet blood film examination, thick/thin blood smear examination, Card Agglutination Test (CATT), ELISA, PCR detection. The available serological tests do not clearly differentiate between *T. evansi*, *T. equiperdum* and *T. brucei* infection due to lot of structural and functional homology. Since these three species are closely related, it is very important to find specific markers for each species to be able to distinguish between them and provide reliable diagnostic tests for timely treatment.

Here we report the first proteomic analysis of *T. evansi* obtained from naturally infected camel which was passaged and purified from mouse blood. Despite unavailability of *T. evansi* genome data our MS based proteomic analysis allowed us to identify more than 100 gene products expressed by the parasite during infection in the rodent model. In addition to providing a glimpse into the biochemical pathways operational during infection, our study has also revealed potential diagnostic markers and possible drug or vaccine candidates against this neglected disease.

## Methods

### Culturing of *T. evansi* and purification of parasites from host

This study was conducted adhering to the institution's guidelines for animal husbandry at National Research Centre on Equines at Hissar. The study was also approved by the institutional review board and ethics committee at National Research Centre on Equines. *T. evansi* camel isolate was maintained *in vivo* using Swiss albino mice through serial passage in the laboratory. At high parasitaemia, blood was collected from tail and subsequently mixed with 0.5–0.8 ml Alsever's solution and inoculated intraperitoneally into healthy rodents. The inoculated rodents were examined 24 hour onwards daily for the presence of parasites and sub inoculated further in fresh rodents; accordingly the strain was maintained.

Purification of host cell free *T. evansi* parasites from rodents/equines are essentially needed for proteomic studies and antigen characterization, which were achieved by the method [10] with minor modifications. For bulk harvest of parasites, 5–10 gm of pre swollen DEAE cellulose (Whatman) was suspended in 200 ml PBS pH 8.0 and allowed to equilibrate for 1 hour at room temperature. The slurry was there after allowed to settle for 30 minutes and supernatant containing the fine granules discarded till the floating granules disappeared from supernatant. The equilibrated suspensions of DEAE cellulose were stored at 4°C until further use.

Cellulose beads were packed into the column of 22 cm length and 3 cm diameter and equilibrated with Phosphate Saline Glucose (PSG) buffer pH 8.0. Infected blood was collected in an anticoagulant (heparin 10 IU/ml) and diluted 1∶1 with PSG buffer and charged through the sides of the column. Column elutes were examined under microscope to spot the purified trypanosomes. Chromatography was performed at room temperature and the parasites were then pelleted after washing thrice with PBS, pH 7.2 and pellet was kept at −80°C for further use.

### Protein extraction and in-gel trypsin digestion for Liquid Chromatography (LC)/MS/MS analysis

Enriched fraction containing 10^8^ to 10^9^ Trypanosomal cells were used for proteomic analysis. The proteins were extracted from enriched parasites by boiling in SDS sample buffer and resolved on an SDS PAGE gel. The proteins were visualized by Coomassie Brilliant Blue (CBB) staining following which the entire lane was excised into 26 pieces. Peptides were extracted by in-gel tryptic digestion from each gel piece. Briefly, proteins from each gel piece were first reduced using DTT and alkylated by iodoacetamide. The modified proteins were then digested with trypsin. The digested peptides were then extracted from the gel using 5% formic acid, 60% Acetonitrile. These peptide mixtures were dissolved in solvent (2% acetonitrile and 98% water containing 0.5% formic acid) and further fractionated by Reverse Phase chromatography on C-18 material on a nano- HPLC system connected online to a nanospray ESI hybrid Q-TOF mass spectrometer from Applied Biosystems. LC-MS-MS experiments were carried out for each sample containing the complex mixture of peptides. A 100 µm ID, 5 µm particle size, 100 Å porosity Michrom column was used for chromatographic separation. The RP chromatography runtime was set for 144 minutes with a flow rate of 300 nL/minute. The mobile phase was adjusted to an increasing concentration of acetonitrile from 5% to 90%, to elute peptides from the column on the basis of their hydrophobicity.

### Data Acquisition using Analyst QS software

Analyst QS software was used to systematically acquire the TOF MS and MS/MS data for each precursor ion entering the instrument from the nanoLC. Eluted peptides were analyzed by one full MS scan and four consecutive product ion scans of the four most intense peaks, using rolling Collision Energy and Dynamic Background Subtract function. An Information Dependant Acquisition (IDA) experiment was used to specify the criteria for selecting each parent ion for fragmentation which included selection of ions in m/z range: >400 and <1600, of charge state of +2 to +5, exclusion of former target ions for 30 seconds, accumulation time of 1 second for a full scan and 2 seconds for MS/MS, ion source voltage of 2200 V and temperature 120°C. MS data for each sample was collected using such user defined criteria for 144 minutes to accommodate the Nano-LC gradient program. The data generated by the Analyst software was stored in a .wiff format.

### Data interpretation using Protein Pilot 2.0 and Global Proteome Machine (GPM)

The original data files were analyzed using the Protein Pilot 2.0 software from a combined database (Swiss Prot 2005, TrEMBL, NCBI and PDB). Peptide scores above 50 and a protein score of minimum 1.3 corresponding to a confidence level greater than 95% were used. GPM analysis tool with mammalian database combined with protozoa was used for peak lists of MASCOT Generic Format generated from the original data file. The error tolerance was set at 100 p.p.m (ESI-QTOF). Spectra of each peptide used for identification of the proteins were verified manually. Presence of at least 5 consecutive y ions or b ions or a combination of both was considered to be significant. The result presented is a combination of 3 independent experiments from mice. Data from each independent experiment was combined and discussed.

## Results

### Identification of *T. evansi* proteins using LC MS/MS

**Figure 1:**
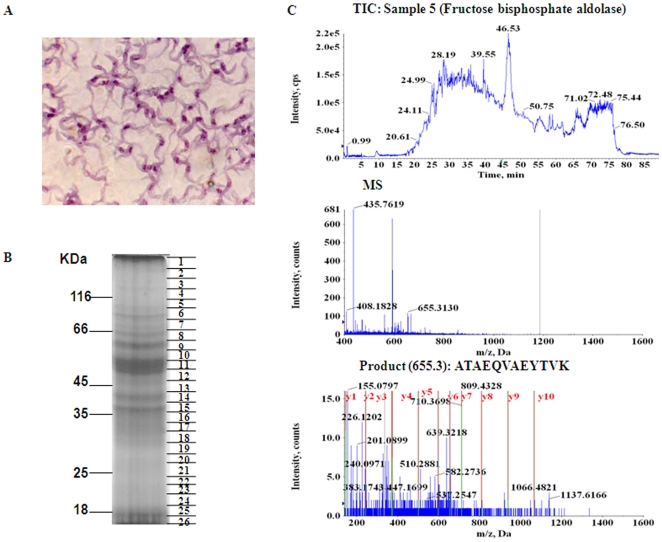
Summary of the protocol followed for MS analyses of samples. **A**. Purified parasites observed at 40× magnification. **B**. Parasites were lysed as described in methods and proteins were fractionated on 10% SDS PAGE. **C**. Individual gel slices were processed as stated in methods and total ion count, MS and MS/MS spectra for Fructose bisphosphate aldolase is shown as an example (from top to bottom respectively).

To examine the proteome of *T. evansi*, we isolated parasites from an infected camel with high parasitaemia and passaged in mice. The mice were then sacrificed and parasites were purified from the blood using ion exchange chromatography as stated in methods. Further, the purified parasites were lysed and protein extraction was carried out as described under methods. One dimensional SDS PAGE was used to resolve the parasite proteins based on their molecular weights and the protein bands were visualized by CBB staining. As can be seen from Figure 1 the most intense bands reproducibly appeared in the molecular weight region between 66 kDa and 45 kDa. We carried out shot-gun proteomics to identify expressed proteins. The entire lane from the SDS-PAGE was excised into 26 contiguous gel slices (Figure 1) and each gel slice was individually processed for in-gel trypsin digestion as described under methods. The peptides obtained from each band were eluted and subjected to nano-LC based RPLC, interfaced with Hybrid Mass Spectrometry. The peak list which included TOF-MS and MS/MS was analyzed by ProteinPilot2.0 software for protein identification. This led to the identification of 166 proteins based on their homologs in *Trypanosoma* as well as other related species whose genome sequences are available.

### Functional categorization reveals an abundance of glycolytic enzymes

**Figure 2:**
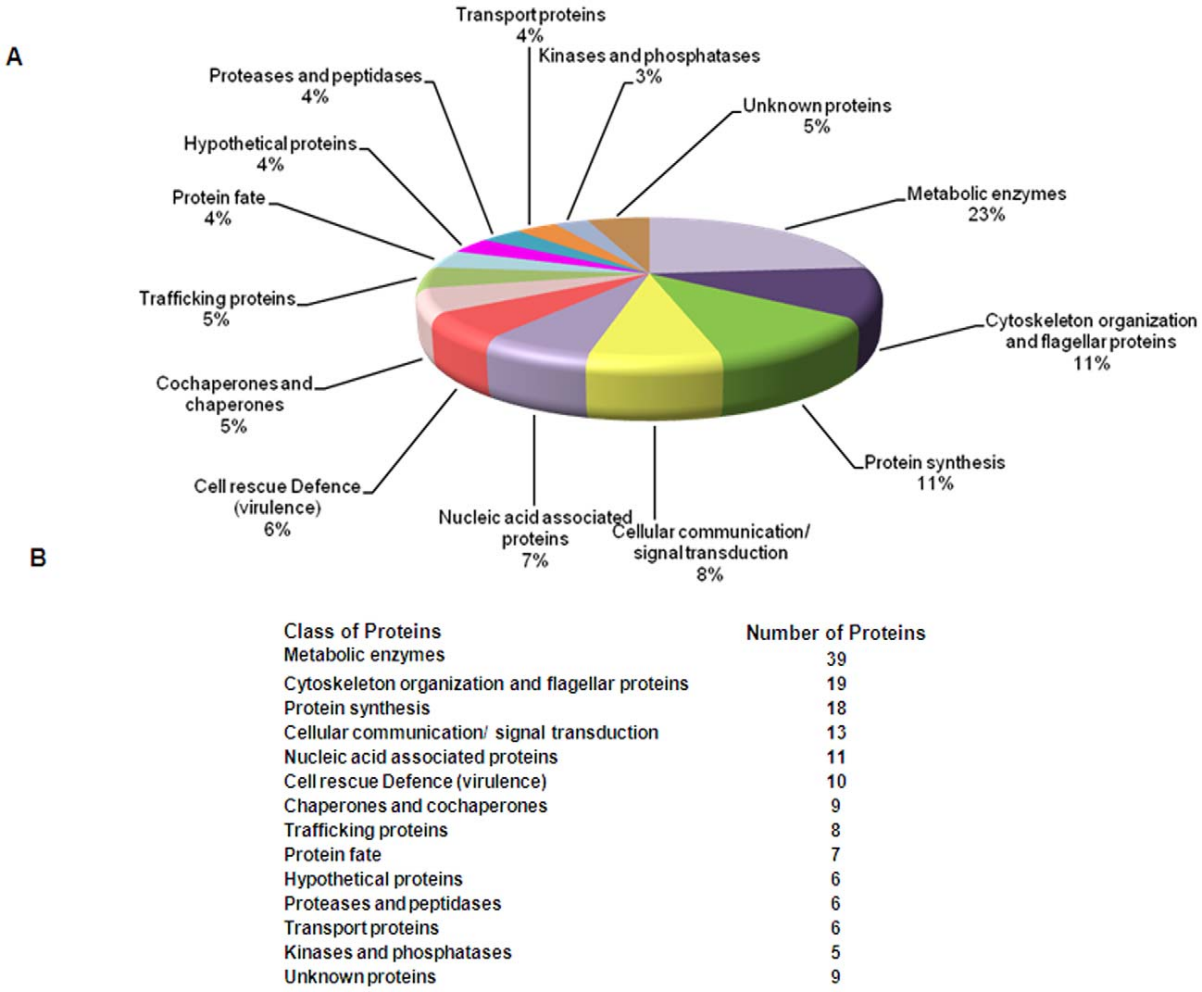
Functional classifications of identified proteins. **A**. Pie chart showing different functional classes of proteins which includes metabolic enzymes, cytoskeletal proteins, proteins involved in synthesis, signal transduction proteins, nucleic acid associated proteins, protein involved in virulence, chaperones and co-chaperones, proteins involved in deciding protein fate, proteins involved in trafficking, hypopthetical proteins, proteases and peptidases, transport proteins, kinases and phosphatases and proteins with unknown functions. **B**. The numbers of proteins present in each functional category have been indicated.

Proteins identified as above were classified according to their functions as shown in Table 1. Details of the protein identifications including their score and number of peptides obtained on data analysis have been listed in Table S1. Metabolic enzymes constituted about 23% of total proteins identified as represented in Figure 2. Among these, enzymes of the glycolytic pathway were most conspicuous, suggesting their abundance and possibly dependence of the parasite on this pathway as the main source of energy. Proteins involved in nucleic acid metabolism, amino acid metabolism, fatty acid metabolism and lipid biosynthesis were also identified from the Trypanosomatids purified from the blood. Interestingly, enzymes involved in TCA cycle could not be detected. This could be due to their lower abundance in the proteome. Cytoskeletal proteins which are generally known to be abundantly present were readily detected. In addition flagellar proteins were also abundantly expressed and identified. Proteins involved in translation as well as main components of Hsp90 chaperone machinery, including Hsp70, DnaJ and cyclophilins were identified. Various isoforms of histone proteins along with other nucleic acid binding proteins were also detected. Proteins involved in cell defence including variable surface glycoproteins and peroxidases were identified. Proteasomal proteins and ubiquitin proteins involved in deciding protein fates were detected.

**Table 1:** Functional classification of identified proteins.

Functional Category	Identified proteins
Metabolic enzymes	
Carbohydrate metabolism	Enolase (EC 4.2.1.11)
	Fructose-bisphosphate aldolase.(aldolase)
	G3PC_TRYBB Glyceraldehyde-3-phosphate dehydrogenase, cytosol.
	Glucose-6-phosphate isomerase.(G6PI)
	Glyceraldehyde-3-phosphate dehydrogenase, glycosomal.(GAPDH)
	Glycerol-3-phosphate dehydrogenase [NAD+], glycosomal.(GPD)
	Glycosomal glyceraldehyde phosphate dehydrogenase (Fragment)
	2,3-bisphosphoglycerate-independent phosphoglycerate mutase (PGAM)
	6-phosphogluconate dehydrogenase (6PGD)
	6-phosphogluconolactonase (6PGL)
	Triosephosphate isomerase, glycosomal.
	Fructose-1,6-bisphosphatase (F1,6,BP)
	Hexokinase.
	Phosphoglycerate kinase.(PGK)
	Pyruvate kinase 1 (PK1)
	6-phospho-1-fructokinase.(PFK1)
	Pyruvate kinase 2 (PK2)
Nucleic acid metabolism	Inosine-5′-monophosphate dehydrogenase (IMPDH)
	Ribonucleoside-diphosphate reductase small chain (RNR2)
	Hypoxanthine-guanine phosphoribosyltransferase (HGPRT)
	Nucleoside diphosphate kinase (NDK)
	Inosine-adenosine-guanosine-nucleoside hydrolase
	Adenylate kinase (ADK)
Amino acid metabolism	S-adenosylhomocysteine (SAH) hydrolase (EC 3.3.1.1)
	Aspartate aminotransferase (AST)
	Branched-chain amino acid aminotransferase, putative (BCAT)
	Alanine aminotransferase, probable (EC 2.6.1.2) (ALT)
	L-threonine 3-dehydrogenase (EC 1.1.1.103)
	Arginine kinase (ARK)
Lipid metabolism	Acyl-CoA synthetase 5
	Fatty acyl CoA synthetase 3.
	Glycerol kinase, glycosomal (GK)
	GIM5A protein
Miscellaneous enzymes	Pyridoxine/pyridoxal/pyridoxamine kinase (PdxK)
	Alternative oxidase, mitochondrial precursor.
	Cytosolic malate dehydrogenase.(MDH)
	Vacuolar ATP synthase catalytic subunit A.
	Trypanothione reductase.
	Trypanothione synthetase.
Cytoskeleton organization and flagellar proteins	Actin 1
	Actin 2
	Alpha tubulin
	Beta tubulin
	69 kDa paraflagellar rod protein (69 kDa PFR protein)
	73 kDa paraflagellar rod protein (73 kDa PFR protein)
	Paraflagellar rod protein
	Paraflagellar rod protein 1
	Flagellar calcium-binding protein TB-1.7G (Fragment)
	Par3
	Valosin-containing protein homolog (VCP)
	I/6 autoantigen
	Axoneme central apparatus protein, possible
	Microtubule-associated protein p320.(MAP320)
	Cytoskeleton-associated protein CAP5.5
	Calflagin Tb-44A.
	Open reading frame A, partial cds. (Fragment)
	Antigen GM6 (Fragment)
Protein synthesis	40S ribosomal protein S14
	40S ribosomal protein S4
	40S ribosomal protein S8
	60S ribosomal protein L10a
	60S ribosomal protein L23a (L25)
	60S ribosomal protein L4 (L1)
	ADP-ribosylation factor 1
	Elongation factor 2 (EF2)
	Ribosomal P0 subunit protein (RPP0)
	Ribosomal protein L24
	Ribosomal protein S12
	EIF-4A
	QM-like protein
	Acidic ribosomal protein P0
	Eukaryotic peptide chain release factor subunit 1(eRFI)
	Ribosomal protein L3
	Elongation factor 1-alpha (EF1-alpha)
	Elongation factor 1 gamma (EF1-gamma)
Cellular communication/signal transduction	14-3-3 protein I
	14-3-3 protein II
	Cyclic nucleotide phosphodiesterase (PDE)
	Cyclic nucleotide-specific phosphodiesterase PDE2A
	Guanine nucleotide-binding protein beta subunit-like protein (GNB2L1)
	Glycosylphosphatidylinositol-specific phospholipase C(GPI-PLC)
	Phospholipase A1, possible (PLA1)
	GTP binding protein, putative (GTR1)
	Adenylyl cyclase (AC)
	Lysophospholipase
	Regulatory subunit of protein kinase A (PKAr)
	CAMP specific phosphodiesterase
Nucleic acid associated proteins	Histone D = CORE histone H4 homolog (Fragments)
	Histone H2B (O96761)
	Histone H3, probable
	Histone H4, putative
	Mitochondrial RNA-binding protein RBP38
	Tcc2i18.9.
	RNA binding protein La-like protein.
	TbRRM1
	RNA binding protein (Rpn)
	Argonaute-like protein 1
	Poly(A) binding protein I (PABP1)
Cell rescue Defence (Virulence)	75 kDa invariant surface glycoprotein precursor
	Variable surface glycoprotein (VSG) (trm|Q26840)
	Variant surface glycoprotein (trm|Q968M4)
	Variable surface glycoprotein (Q968M5)
	Variable surface glycoprotein (Q6QA67)
	Variant surface glycoprotein precursor (trm|Q26841)
	Non-variant surface glycoprotein (Fragment)
	Tryparedoxin peroxidase
	H25N7.12 protein
	IgE-dependent histamine-releasing factor, putative (HRF)
Chaperones and cochaperones	Chaperonin HSP60, mitochondrial precursor
	Cyclophilin A (CypA)
	DnaJ protein, putative
	Heat shock 70 kDa protein 4 (HSP70) (spt|P11145)
	Heat shock protein 83
	Mitochondrial HSP70
	BiP/GRP78 precursor
	P69 antigen
	TcSTI1
Protein fate	Proteasome subunit alpha type 1
	Lysosomal/endosomal membrane protein p67.
	Proteasome regulatory non-ATP-ase subunit 5
	Ubiquitin
	Ubiquitin-conjugating enzyme E2
	Proteasome subunit alpha type 5(PSMA5)
	Proteasome subunit beta type 3 (PSMB3)
	20S proteasome alpha 7 subunit(PSMA7)
Proteins involved in trafficking	Dynamin-related protein (DRP1)
	Gpi8 transamidase precursor
	ESAG5
	Putative coatomer beta subunit (COBP)
	Transferrin-binding protein (TBP)
	Soluble N-ethylmaleimide sensitive factor (NSF) attachment protein possible
	Adaptor gamma-1 chain (AP1G1)
	Clathrin heavy chain (CHC)
	Rab1
Hypothetical proteins	Hypothetical protein (trm|Q7YVL1)
	Hypothetical protein (trm|Q7YSV0)
	Hypothetical protein (Q7YVJ5)
	Hypothetical protein Tb10.70.1130 (XP_822825.1)
	Hypothetical protein Tb09.211.3955(XP_827537.1)
	Hypothetical protein, conserved (XP_844790.1)
Proteases/Peptidases	Cysteine protease
	Cysteine proteinase precursor
	Oligopeptidase A
	Oligopeptidase B
	Calpain-like protein, probable (CAP)
	Metacaspase
Transport Proteins	Rhodesiense ADP/ATP carrier
	P-type H+-ATPase
	Aquaglyceroporin 3 (AQP3)
	Calcium motive P-type ATPase TBCA1 (Fragment)
	Probable biopterin transporter (Esag10)
	Pentamidine resistance protein (PRP)
Kinases and phosphatases	Acidic phosphatase (AP)
	Casein kinase 1 homolog 1(CK1)
	C-terminal kinesin KIFC1
	Protein kinase CK2 alpha
	Protein phosphatase 2A catalytic subunit (PP2A)
Unknown functions	Bloodstream-specific protein 2 precursor
	Bloodstream-specific protein 1.3-4.
	Glycosomal membrane protein
	Laminin receptor-like protein/p40 ribosome associated-like protein
	RHS6a
	Transmembrane glycoprotein
	Retrotransposon hot spot protein, RHS4l
	RHS4a (Retrotransposon hot spot protein RHS4-a
	Retrotransposon hot spot protein RHS4-b

Relatively few organellar proteins or secretory proteins were detected suggesting that the parasite may not be very active in protein traffic related functions during growth in the bloodstream. Proteases, peptidases protein, kinases and phosphatases previously implicated as potential drug targets were also detected. The list of proteins identified also included 6 hypothetical proteins which were homologous to proteins from *T. brucei*.

**Figure 3:**
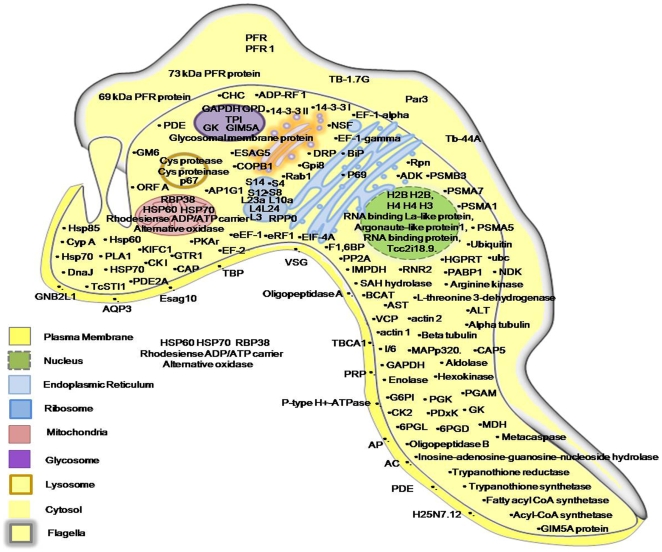
Representation of proteins according to their cellular localization in *T. evansi*. All the proteins identified have been categorized based on their homology to related Trypanosomal species and their known localizations in those species.

Furthermore, based on homology of the identified proteins to other Trypanosomal species and their known localizations in those species, we have categorized the proteins according to their presence in different cellular compartments as shown in Figure 3. We have also attempted to classify the identified proteins on the basis of their solubility. Table S2 provides a list of probable membrane bound and soluble proteins from a total of 166 identified proteins. The membrane proteins possibly constitute those which are associated with organellar membranes or the plasma membrane. Additionally, we have also detected post translational modifications in some proteins amongst which acetylations and phosphorylations were the most common modifications observed. Table S3 provides a list of the identified proteins having PTMs along with their MS/MS spectra which is shown in Figure S1. It is interesting to note that the identified proteins constitute those which are involved in host pathogen interactions including potential surface antigens and virulence factors from parasites. Indeed many of the proteins identified were known drug targets or vaccine candidates in related trypanosomal species.

## Discussion

Over the past few years there is an increasing emphasis on examination of parasite pathways prevalent during disease manifestation within the host. There is growing awareness that some of these pathways could be significantly present only during host pathogen interactions often missing in lab grown cultures. Proteins unique to clinical samples could thus prove to be keystone drug targets.

In recent times a neglected animal disease called surra caused by *T. evansi* has lead to significant economic losses to livestock industry. While traditionally *T. evansi* infections have been observed in domestic and wild animals, recent reports suggest their ability to infect humans [2]. Currently available treatments have many toxic side effects and there is an urgent need to identify better treatment strategies. *T. evansi* is the least studied parasite among all the Trypanosomatids. Proteome studies of *T. cruzi*, *T. brucei* and *Leishmania major* have been carried out in the recent years revealing several interesting features of the parasite lifecycle [11]–[17]. In *T. cruzi* a total of 396 proteins were identified by LC-MS/MS from epimastigotes [11]. All these studies had been carried out using parasites cultured *in-vitro*. In this study, we report the proteomic analysis of *T. evansi* from camel which was passaged in mice.

Using mass spectrometry based proteomics approaches we have developed a snapshot of the proteome of trypanosomatid stage in the blood stream during infection in camel. Based on homology driven searches, we have identified 166 proteins of *T. evansi*. The most abundant proteins identified were the metabolic enzymes in which enzymes involved in the glycolytic pathway constituted a major class. Out of the 40 metabolic enzymes identified, 17 enzymes were of the glycolytic pathway. Glycolysis has been shown to be the main source of ATP generation in bloodstream stages in *T. brucei* as well [18]. Infact glycolytic enzymes of *Trypanosoma* have been proposed as attractive drug targets. Indeed characteristic flagellar motion of *T. evansi* is known to depend on glucose concentration in the medium. Enzymes of the TCA cycle were conspicuously absent in our study. Trypanosomes have been shown to rely on mitochondrial metabolism only in the insect vector since the absence of kDNA has been shown to hamper mitochondrial development. *T. evansi* which lacks maxi-circle kDNA relies on glycolytic enzymes as their main energy source.

Among the other proteins identified, there were many potential diagnostic markers, drug targets as well as possible vaccine candidates. These included cytoskeletal proteins such as two actin proteins [19], two paraflagellar rod proteins [20] and calflagin [21], Metabolic enzymes including trypanothione reductase [22], tryparedoxin peroxidase [23], trypanothione synthetase [24], kinases such as casein kinase 1 [25], chaperone proteins such as heat shock like 85 kDa protein [26], signal transduction proteins like glycosylphosphatidylinositol-specific phospholipase C [27], proteases and peptidases such as cysteine protease [28], oligopeptidase B [29], trafficking proteins such as GPI 8 transamidase [30], transport proteins such as aquaglyceroporin 3 [31] and calcium motive P-type ATPase [32]. Having obtained partial sequences of these proteins future studies towards cloning their genes and molecular characterisations may be possible.

While actin is a ubiquitously expressed conserved cytoskeletal protein, a recent study showed that recombinant *T.evansi* actin expressed in *Esherichia coli* could be a potential vaccine candidate. Immunization of animals with actin provided immunity against 3 species of Trypanosomal infections, namely *T.evansi*, *T.equiperdum* and *T.brucei*. This cross reactivity is due to the extensive homology shared by the actin of these three species [19]. Recently around 90 different GPI (glycosylphosphatidylinositol)-species were identified from lab grown cultures of *T. cruzi* by an LC-MS based approach [33]. We have identified four different VSG's. Furthermore, proteins involved in the shedding of the GPI anchored VSG such as ESAG5 and GPI specific phosholipase C were also identified. There are reports which suggest sequence similarity of GPI specific phospholipase C and bacterial phospholipase C. It would be interesting to also look at the GPI-moieties of these VSG's since they are known to be potent immunogens [34]. *T. evansi* are known to exhibit a typical lashing action of the powerful locomotory flagella which results in mechanical injury to erythrocytes and other cells in the blood leading to anaemia. We have identified Calflagin a known calcium binding protein localized in the flagella of the parasite. Calflagin has been shown to act as a calcium sensor and modulate the flagellar mobility [35]. Also the C terminal fragment of the calflagin was recommended as a probable candidate for serological detection of *T. cruzi* in humans [21]. Furthermore paraflagellar rod proteins which were detected in our study are currently being investigated as potential vaccine candidates in *T. cruzi* [36], [37]. It has been reported that these proteins were purified from *T. cruzi* epimastigotes and used to immunize mice against trypanosomal infections [20].

Among the potential drug targets, tryparedoxin peroxidase is a glutathione peroxiredoxin, unique to trypanosomes. This enzyme detoxifies peroxynitrite radicals produced by macrophages and hence plays an important role in successful evasion of host defense system. Our study also identified protein kinases expressed by *T. evansi* during infection. The kinome of *T. cruzi* and *T. brucei* have been studied and novel chemotherapeutic agents against these kinases have been suggested [25]. Additionally, we have identified oligopeptidase B in our study. This protein is known to play a major role during parasite invasion into host cell in *T. cruzi* [38]. In *T. evansi* infections Oligopeptidase B has a major role to play in manifestation of disease. It is shown to proteolyticaly cleave many of the host derived peptides and proteins like kinogen, atrial natriuretic factor etc. Inability to inhibit this cysteine peptidase by host derived protease inhibitors makes Oligopeptidase B important during pathogenesis, thus making it an attractive drug target [39].

Overall our study highlights the use of contemporary proteomic approaches to study clinical proteome of *T. evansi*. Observations made in this study are potentially extrapolatable to *T. cruzi*, *T. brucei* infections in humans for which clinical proteomes have not been examined thus far. In addition to providing a glimpse into the cell biology, pathogenesis and metabolic state of the parasite, this study will aid in the development of diagnostic markers, drug targets and vaccine candidates.

## Supporting Information

Table S1 Details of the protein identifications including their score and number of peptides obtained on data analysis.
(0.16 MB XLS)

Table S2 List of proteins classified on the basis of their solubility.
(0.11 MB DOC)

Table S3 List of proteins and their respective peptides along with their post translational modifications.
(0.04 MB DOC)

Figure S1 MS/MS spectra of identified proteins having post translational modifications.
(1.58 MB DOC)
