# Supplementary material for: Proteomics of Trypanosoma evansi Infection in Rodents
Source: PLoS One. 2010 Mar 22;5(3):e9796. doi: 10.1371/journal.pone.0009796 (PMC2842431; doi:10.1371/journal.pone.0009796)
Supplement: Table S2 — List of proteins classified on the basis of their solubility. (0.11 MB DOC) [file pone.0009796.s002.doc]

**Table S2:** **Classification of identified proteins based on their solubility.**

| **Membrane bound** | **Soluble proteins** |
| --- | --- |
| GIM5A protein | QM-like protein |
| Rhodesiense ADP/ATP carrier | Mitochondrial RNA-binding protein RBP38 |
| 75 kDa invariant surface glycoprotein precursor | Proteasome subunit alpha type 1 |
| Variable surface glycoprotein (Q26840) | Dynamin-related protein |
| Variant surface glycoprotein (Q968M4) | C-terminal kinesin KIFC1 |
| Variant surface glycoprotein precursor  (Q26841) | Metacaspase |
| Non-variant surface glycoprotein (Fragment) | Calpain-like protein, probable |
| Vacuolar ATP synthase catalytic subunit A | Tcc2i18.9 |
| Transmembrane glycoprotein | Hypoxanthine-guanine |
| Glycosomal membrane protein | Phosphoribosyltransferase |
| Lysosomal/endosomal membrane protein p67 | Histone D=CORE histone H4 homolog |
| Acidic phosphatase | Histone H2B (O96761) |
| Glycerol-3-phosphate dehydrogenase, glycosomal | Histone H2B (P27795) |
| Alternative oxidase, mitochondrial precursor | Histone H3, probable |
| Fatty acyl CoA synthetase 3 | Histone H4, putative |
| Phospholipase A1, possible | Glycosylphosphatidylinositol-specific phospholipase C |
| Pentamidine resistance protein | Guanine nucleotide-binding protein beta subunit-like protein |
| ESAG5 | Rab1 |
| Unnamed protein product | 14-3-3 protein II |
| Hypothetical protein ( Q7YSV0) | 14-3-3 protein I |
| Aquaglyceroporin 3 | Mitochondrial HSP70 |
| P-type H+-ATPase | Heat shock protein 83 |
| Calcium motive P-type ATPase TBCA1 | Heat shock protein (HSP70) |
| Probable biopterin transporter (Esag10) | Heat shock like 85 kDa protein |
| H25N7.12 protein | Heat shock 70 kDa protein 4 (HSP70) |
| Adenylyl cyclase | Heat shock 70 kDa protein |
| Transferrin-binding protein | DnaJ protein, putative |
| Variable surface glycoprotein (Q968M5) | Cyclophilin A |
| Variable surface glycoprotein (Q6QA67) | Chaperonin HSP60, mitochondrial precursor |
| Hypthetical Protein (XP_822825.1) | Ribosomal protein S12 |
| Hypothetical Protein (XP_844790.1) | 40S ribosomal protein S14 |
| Acyl-CoA synthetase 5 | 40S ribosomal protein S4 |
| Laminin receptor-like protein | 40S ribosomal protein S8 |
| Oligopeptidase A (EC 3.4.24.15) | 60S ribosomal protein L10a |
| Retrotransposon Hot Spot protein 4b (RHS4b) | 60S ribosomal protein L23a (L25) |
| Retrotransposon Hot Spot protein41 (RHS41) | 60S ribosomal protein L4 (L1) |
| Retrotransposon Hot Spot protein 4a (RHS4a) | ADP-ribosylation factor 1 |
| Hypothetical protein (Q7YVJ5) | Elongation factor 2 |
| EIF-4A | Ribosomal P0 subunit protein |
|  | Ribosomal protein L24 |
|  | Valosin-containing protein homolog |
|  | Par3 |
|  | Paraflagellar rod protein 1 |
|  | 73 kDa paraflagellar rod protein |
|  | 69 kDa paraflagellar rod protein |
|  | Inosine-adenosine-guanosine-nucleoside hydrolase |
|  | 6-phospho-1-fructokinase |
|  | 6-phosphogluconolactonase |
|  | Pyruvate kinase 1 |
|  | Poly(A) binding protein I |
|  | Arginine kinase |
|  | Pyridoxine/pyridoxal/pyridoxamine kinase |
|  | Hexokinase |
|  | Fructose-1,6-bisphosphatase |
|  | Alanine aminotransferase, probable |
|  | Aspartate aminotransferase |
|  | S-adenosylhomocysteine hydrolase |
|  | Glycosomal glyceraldehyde phosphate dehydrogenase |
|  | Glycerol kinase, glycosomal |
|  | Glyceraldehyde-3-phosphate dehydrogenase, cytoslic |
|  | Fructose-bisphosphate aldolase |
|  | Enolase (EC 4.2.1.11) |
|  | 6-phosphogluconolactonase (EC 3.1.1.17) |
|  | Tryparedoxin peroxidase |
|  | Cytosolic malate dehydrogenase |
|  | 2,3-bisphosphoglycerate-independent phosphoglycerate mutase |
|  | 6-phosphogluconate dehydrogenase |
|  | Glucose-6-phosphate isomerase |
|  | Nucleoside diphosphate kinase |
|  | Alpha tubulin |
|  | Beta tubulin |
|  | Actin 2 |
|  | Actin 1 |
|  | Glycerolkinase |
|  | I/6 autoantigen |
|  | Axoneme central apparatus protein, possible |
|  | BiP/GRP78 precursor |
|  | P69 antigen |
|  | Gpi8 transamidase precursor |
|  | Cysteine proteinase precursor |
|  | Bloodstream-specific protein 2 precursor |
|  | Flagellar calcium-binding protein TB-1.7G |
|  | Cyclic nucleotide-specific phosphodiesterase PDE2A |
|  | Cysteine protease (Fragment) |
|  | Oligopeptidase B |
|  | Triosephosphate isomerase, glycosomal |
|  | Cyclic nucleotide phosphodiesterase |
|  | RHS6a |
|  | Proteasome subunit alpha type 5. |
|  | RNA binding protein |
|  | Lysophospholipase |
|  | Proteasome subunit beta type 3 |
|  | 20S proteasome alpha 7 subunit |
|  | Calflagin Tb-44A |
|  | Soluble NSF attachment protein possible antigenic protein |
|  | Cytoskeleton-associated protein CAP5.5 |
|  | Ubiquitin |
|  | Regulatory subunit of protein kinase A |
|  | Protein kinase CK2 alpha |
|  | Pyruvate kinase 2 |
|  | RNA binding protein La-like protein |
|  | L-threonine 3-dehydrogenase |
|  | Ribonucleoside-diphosphate reductase small chain |
|  | Protein phosphatase 2A catalytic subunit |
|  | TbRRM1 |
|  | Phosphoglycerate kinase |
|  | Elongation factor 1-alpha |
|  | Acidic ribosomal protein P0 |
|  | Trypanothione reductase |
|  | Eukaryotic peptide chain release factor subunit 1 |
|  | Inosine-5'-monophosphate dehydrogenase |
|  | Proteasome regulatory non-ATP-ase subunit 5 |
|  | GTP binding protein |
|  | Ribosomal protein L3 |
|  | Microtubule-associated protein p320 |
|  | Putative coatomer beta subunit |
|  | TcSTI1 |
|  | Adaptor gamma-1 chain |
|  | Trypanothione synthetase |
|  | CAMP specific phosphodiesterase |
|  | Argonaute-like protein 1 |
|  | Open reading frame A, partial cds |
|  | Histone H2B (P27795) |
|  | Elongation factor 1 gamma |
|  | IgE-dependent histamine-releasing factor |
|  | Antigen GM6 |
|  | Hypothetical protein (XP_827537.1) |
|  | Glyceraldehyde-3-phosphate dehydrogenase, glycosomal. |
|  | Hypothetical protein (Q7YLV1). |
|  | Adenylate kinase |
|  | Clathrin heavy chain |
|  | Probable ubiquitin-conjugating enzyme (UBC) |
