# Supplementary material for: Proteomics of Trypanosoma evansi Infection in Rodents
Source: PLoS One. 2010 Mar 22;5(3):e9796. doi: 10.1371/journal.pone.0009796 (PMC2842431; doi:10.1371/journal.pone.0009796)
Supplement: Table S3 — List of proteins and their respective peptides along with their post translational modifications. (0.04 MB DOC) [file pone.0009796.s003.doc]

**Table S3: List of post translational modifications of proteins and their respective peptides**.

| Sl.no | Accession number. | Protein | Modification | Peptide |
| --- | --- | --- | --- | --- |
| 1 | A45616 | Antigenic protein | N terminal -acetylation | AANEQYETLQR |
| 2 | Q9U420 | Arginine Kinase | N terminal -acetylation | ATRDVAAELEK |
| 3 | Q76NL1 | Alpha tubulin | S - acetylation | LIGQWSSLTAS(Ac)LR |
| 4 | Q94775 | Guanine nucleotide-binding protein beta subunit like – protein | N terminal -acetylation | AVAYEGQLTGHR |
| 5 | Q68CL8 | 14-3-3 protein II | N terminal -acetylation | AGFQIPEKR |
| 6 | U26768 | I/6 Autoantigen | N terminal -acetylation | MLCPPDVAFEKR |
| 7 | Q27787 | Protein phosphatase 2A catalytic subunit. | N terminal -acetylation | MDTIDQFLETVGK |
| 8 | PQ4789 | Triose phosphate isomerase, glycosomal. | N terminal -acetylation | SKPQPIAAANWK |
| 9 | Q7YUN3 | Adenlyate cyclase | N terminal -acetylation | AVLSEDVLLYLK |
| 10 | Q9XZ65 | Laminin receptor- like protein/ p40 ribosome associated like protein | Phoshorylation | SIS[pho]MMYWLLAR |
| 11 | Q968M5 | Variable surface glycoprotein | S- acetylation | VALETVNSIDKLQQALEFYTAR |
| 12 | Q9NG18 | 2.3. bisphosphoglcerate – independent phosphoglycerate mutase. | N terminal -acetylation | ALTLAAHK |
| 13 | Q226840 | Variable surface glycoprotein | N terminal -acetylation | ELGTVTDTAELQK |
| 14 | Q26733 | BiP/GRP78 precursor | T – phosphorylation and N - oxidation | AVVT[Pho]VPAYFN[Oxi]DAQR |
| 15 | Q968M5 | Variable surface glycoprotein | S – acetylation | VILPAVAYGGEVAGAIS[Ac]SALK |
| 16 | Q26789 | 73KDa paraflagellar rod protein | N terminal -acetylation | AAVDDATGLEAAR |
| 17 | P06660 | Heat shock like 85KDa protein | S - phosphorylation | RGVVDSEDLPLNISR |
