# Supplementary material for: Proteomics of Trypanosoma evansi Infection in Rodents
Source: PLoS One. 2010 Mar 22;5(3):e9796. doi: 10.1371/journal.pone.0009796 (PMC2842431; doi:10.1371/journal.pone.0009796)
Supplement: Figure S1 — MS/MS spectra of identified proteins having post translational modifications. (1.58 MB DOC) [file pone.0009796.s004.doc]

**Supplementary Figure S1: MS/MS spectra of identified proteins having post translational modifications.**


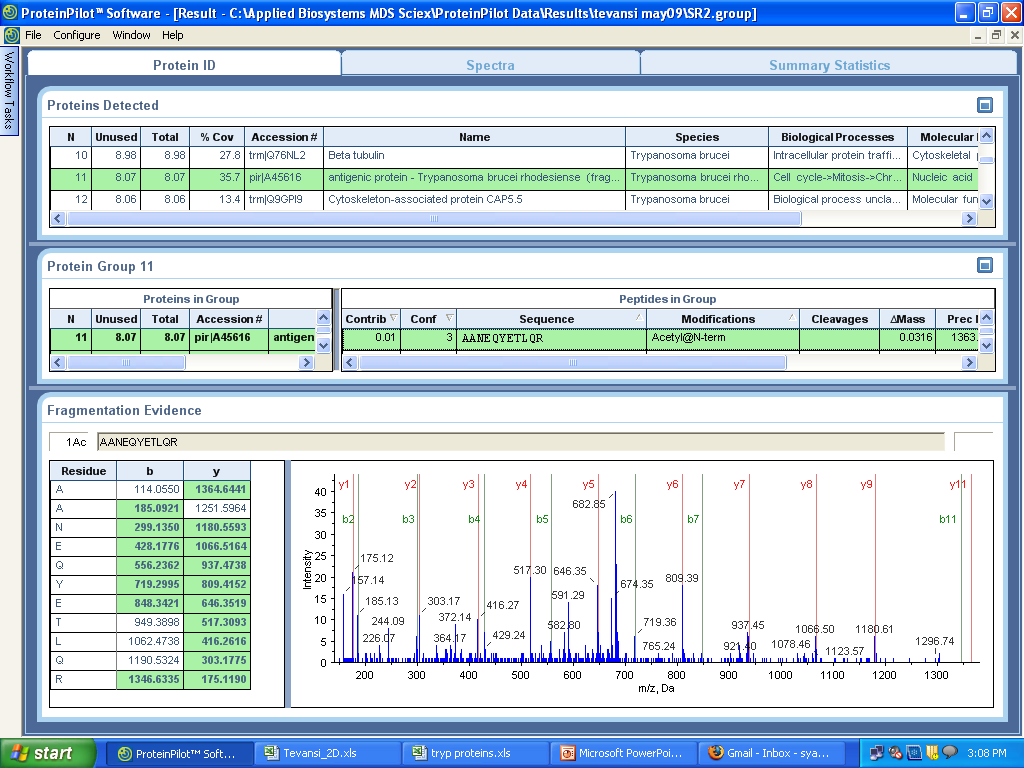


1. AANEQYETLQR : N – terminal acetylation: A45616 : Antigenic protein


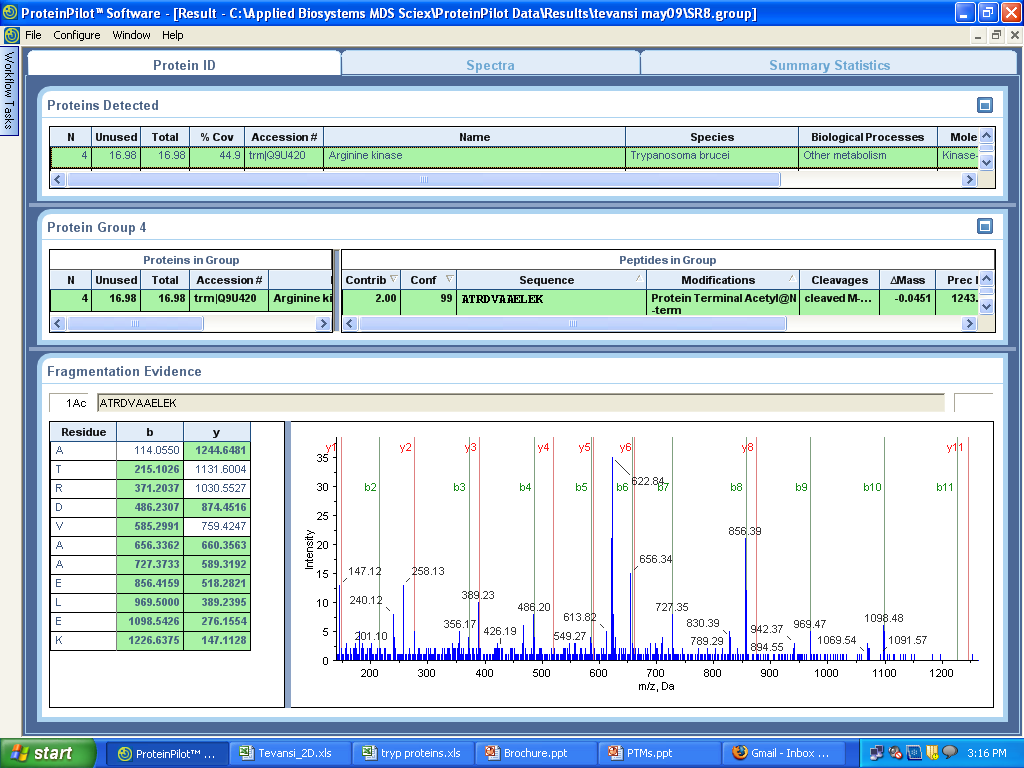


2. ATRDVAAELEK: N – terminal acetylation: Q9U420 : Arginine Kinase


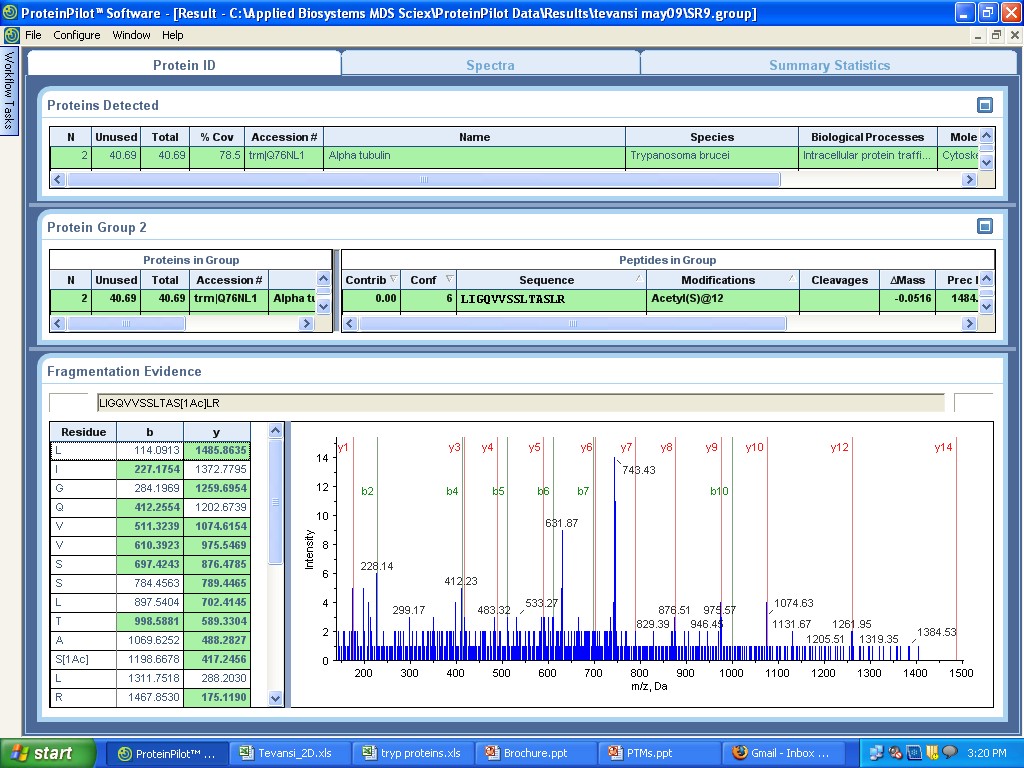


3. LIGQWSSLTAS(Ac)LR: S- acetylation: Q76NL1: Alpha tubulin


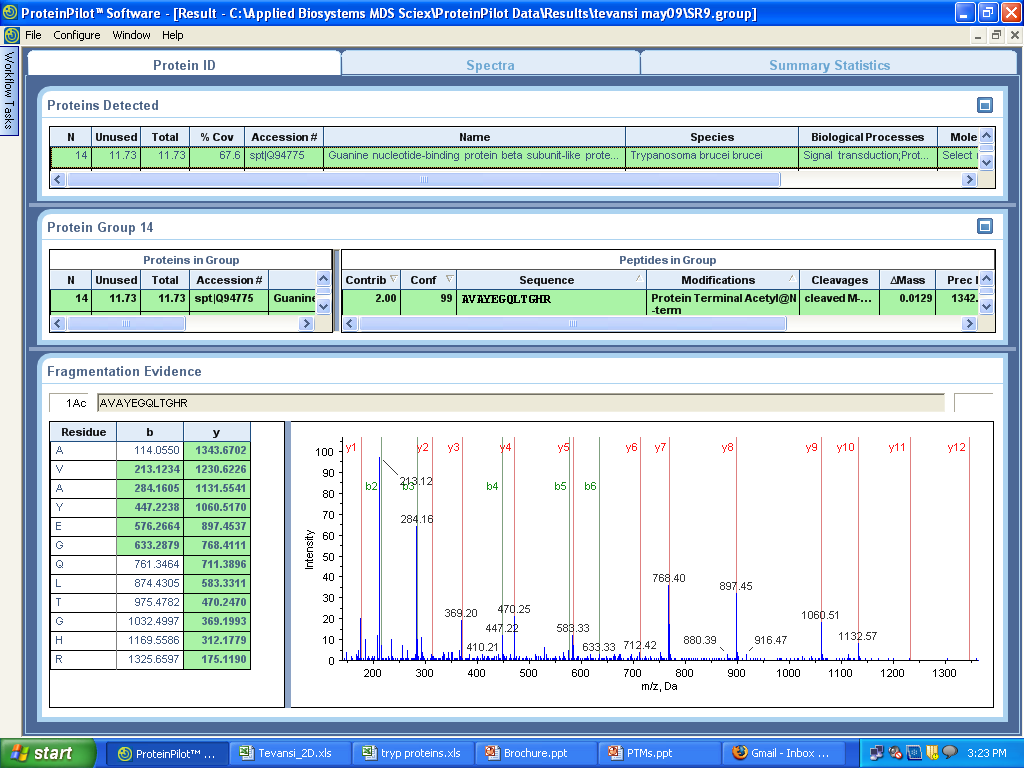


4. AVAYEGQLTGHR: N terminal –acetylation: Q94775:

Guanine nucleotide-binding protein beta subunit like – protein


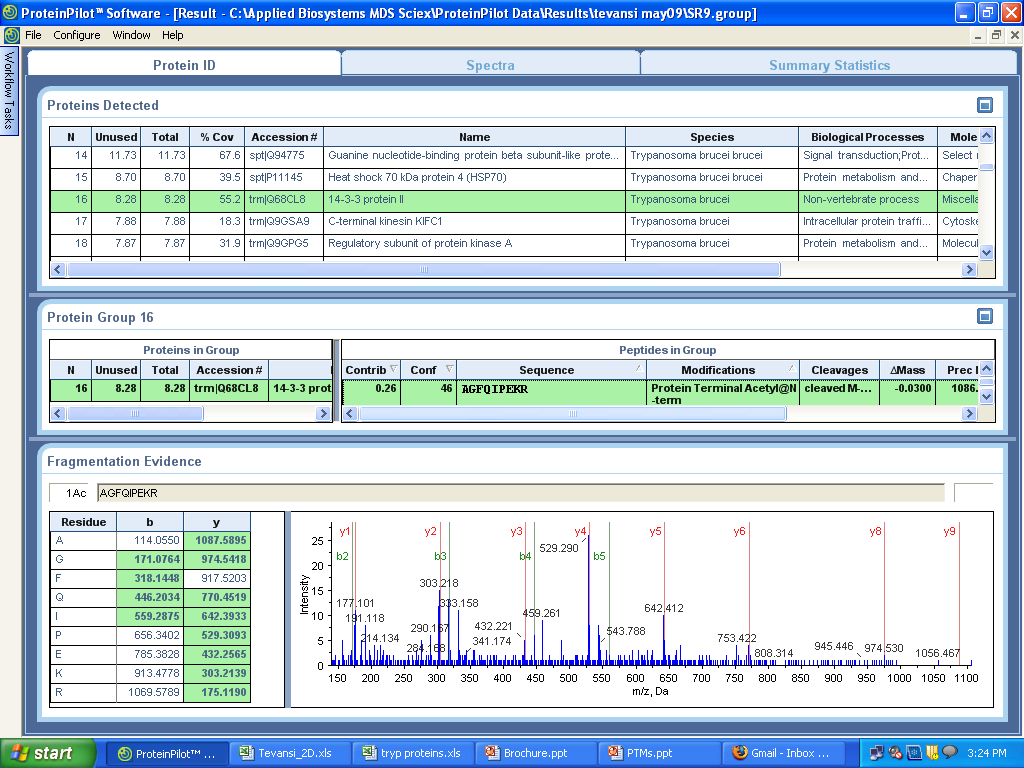


5.AGFQIPEKR: N terminal –acetylation: Q68CL8:14-3-3 protein II


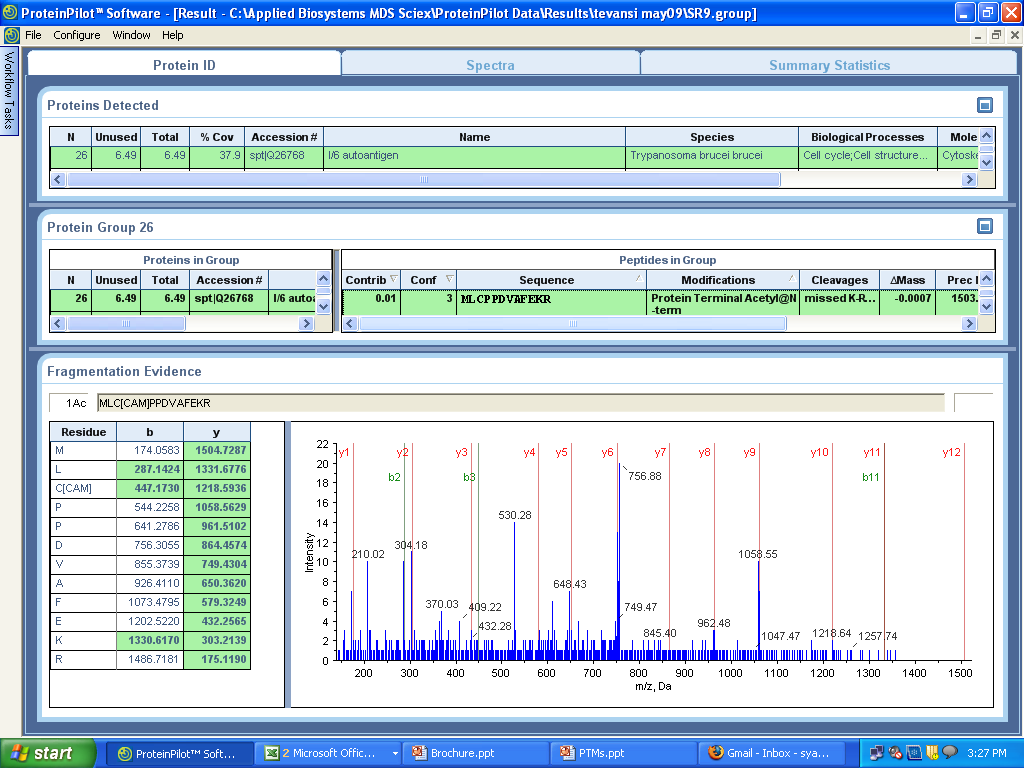


6.MLCPPDVAFEKR: N terminal –acetylation: U26768: I/6 antigen


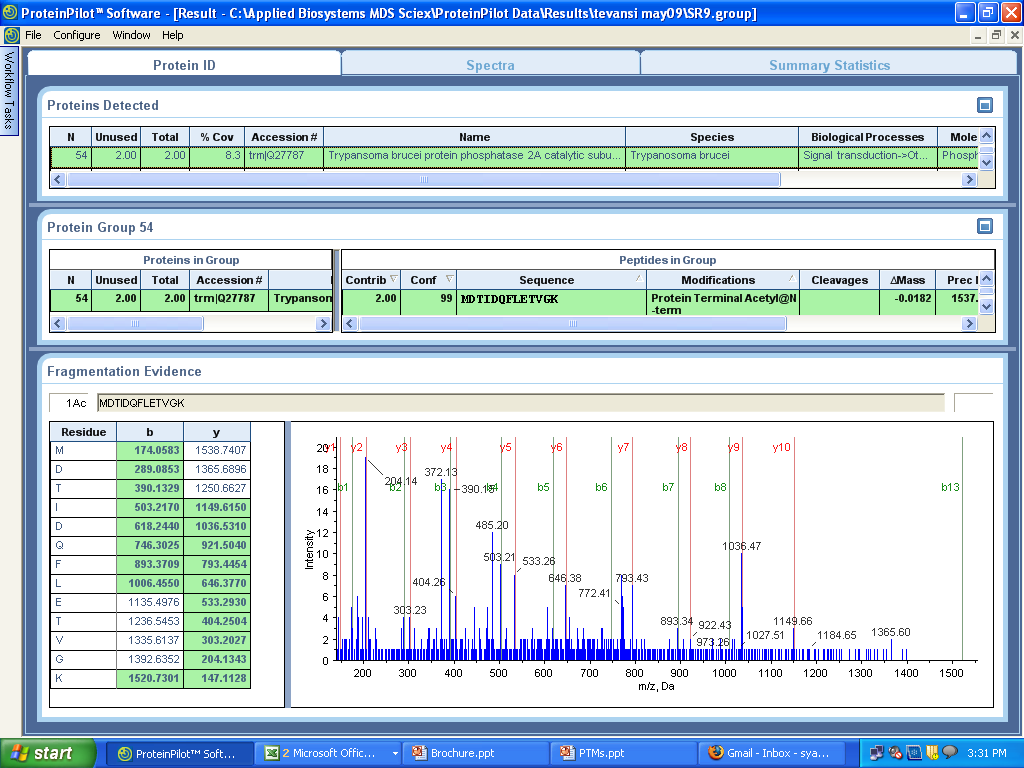


7. MDTIDQFLETVGK: N terminal –acetylation: Q27787: Protein phosphatase 2A

catalytic subunit.


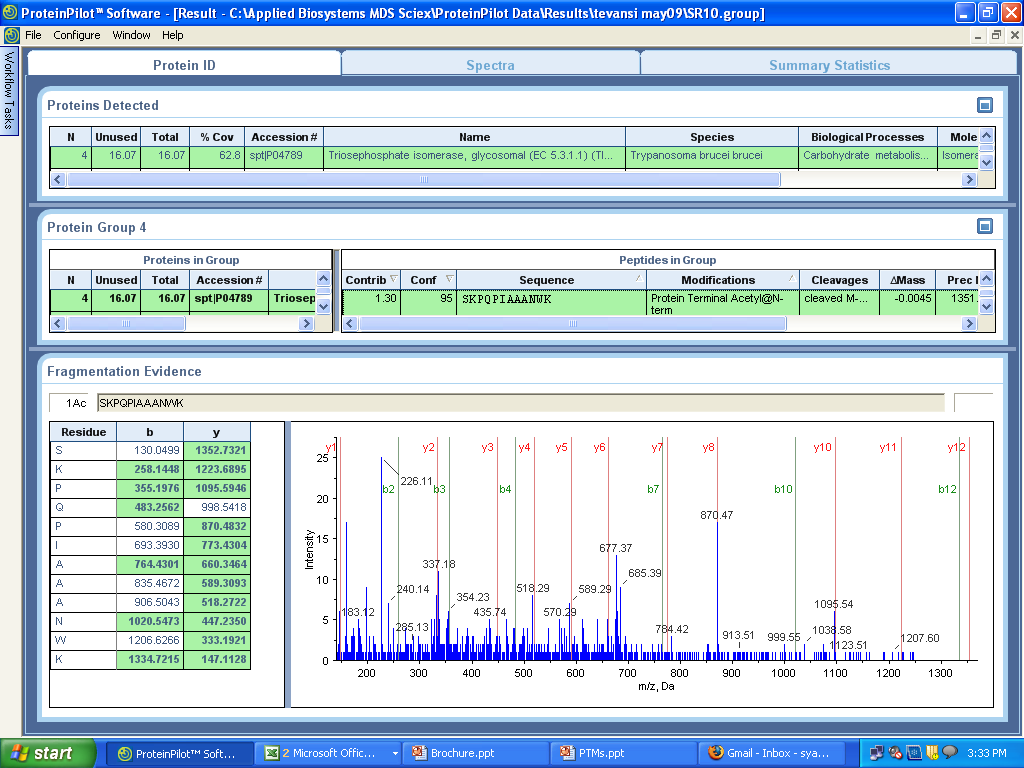


8. SKPQPIAAANWK: N terminal –acetylation: PQ4789: Triose phosphate

isomerase, glycosomal.


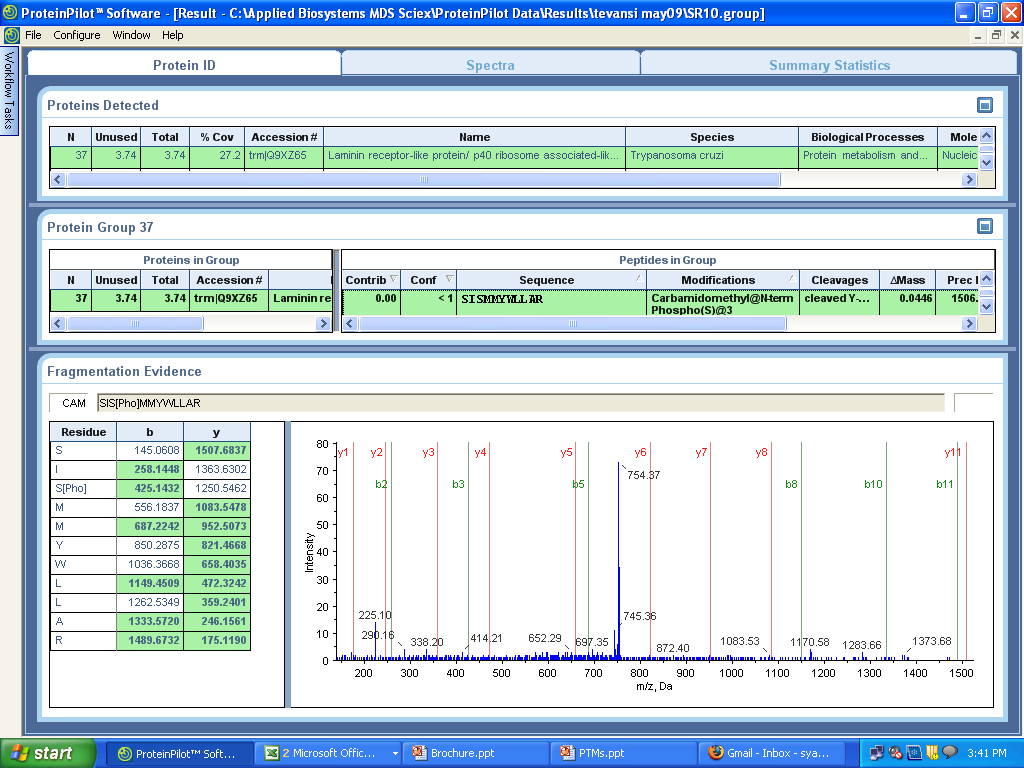


10. SIS[pho]MMYWLLAR: Phoshorylation: Q9XZ65: Laminin receptor- like protein/p40 ribosome associated like protein


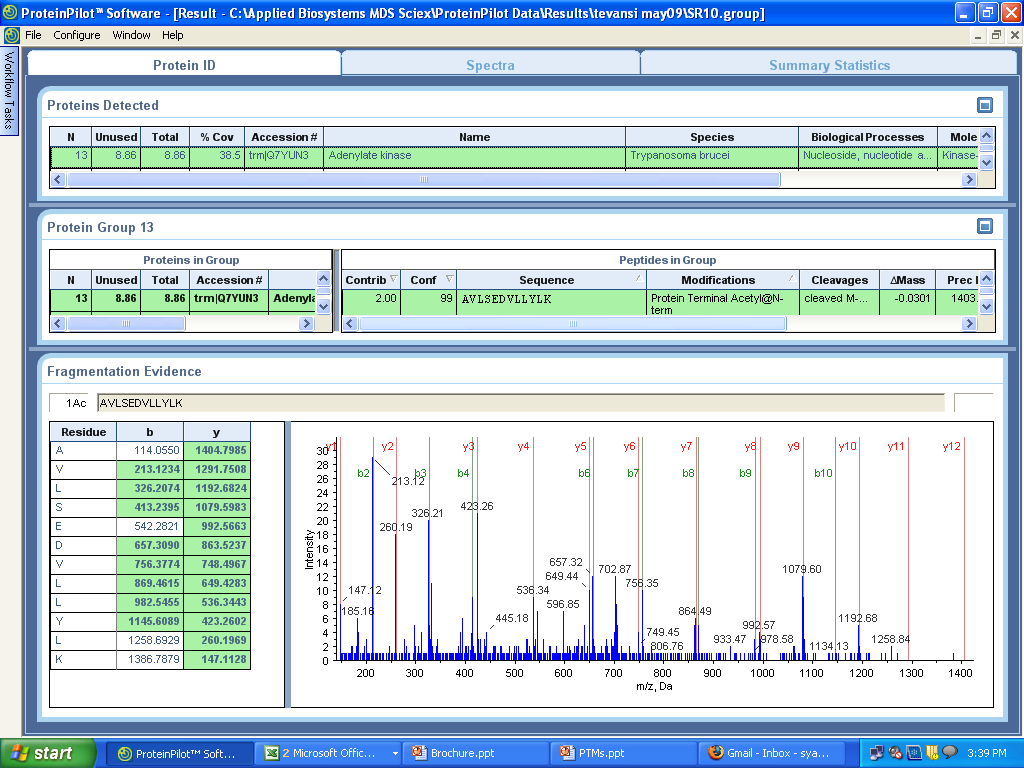


9. AVLSEDVLLYLK: N terminal –acetylation: Q7YUN3: Adenlyate cyclase


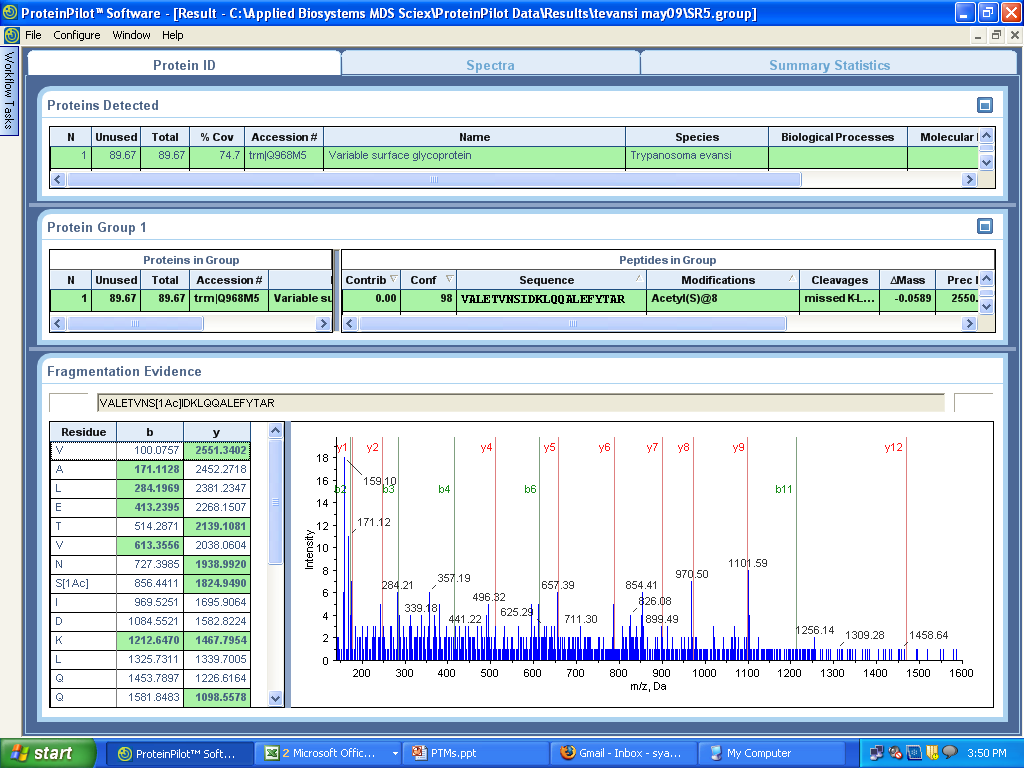


11.VALETVNS[Ac]IDKLQQALEFYTAR: S- acetylation: Q968M5: Variable

surface glycoprotein


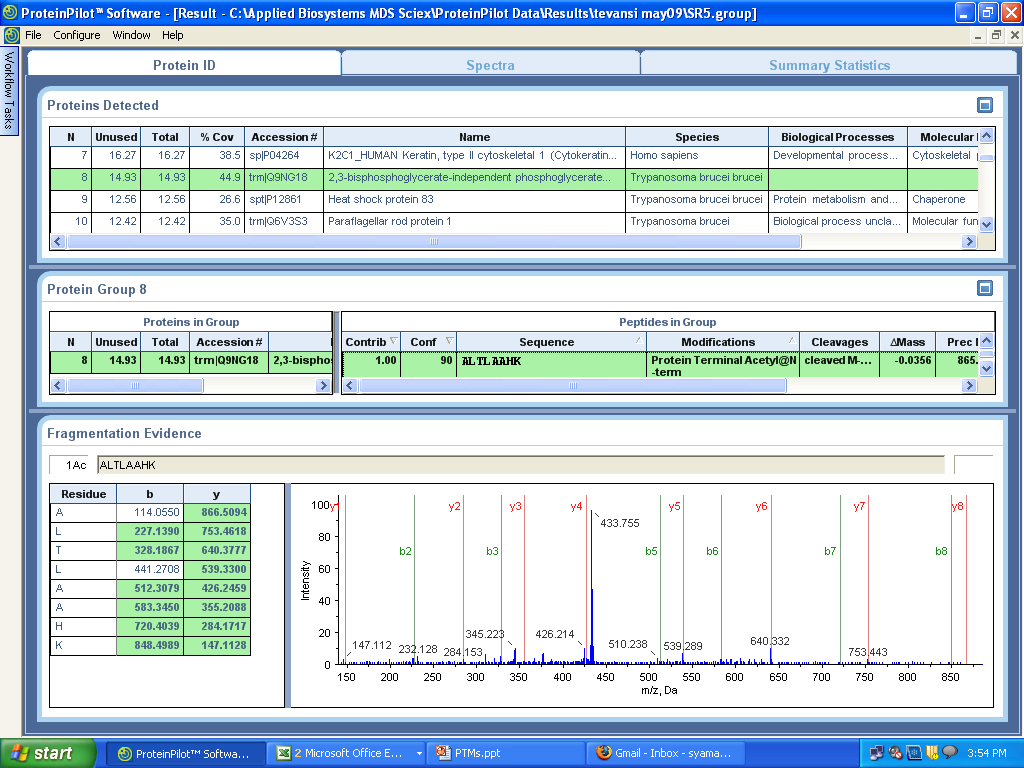


12. ALTLAAHK :N terminal –acetylation: Q9NG18:

2,3-bisphosphoglycerate-independent phosphoglycerate mutase.


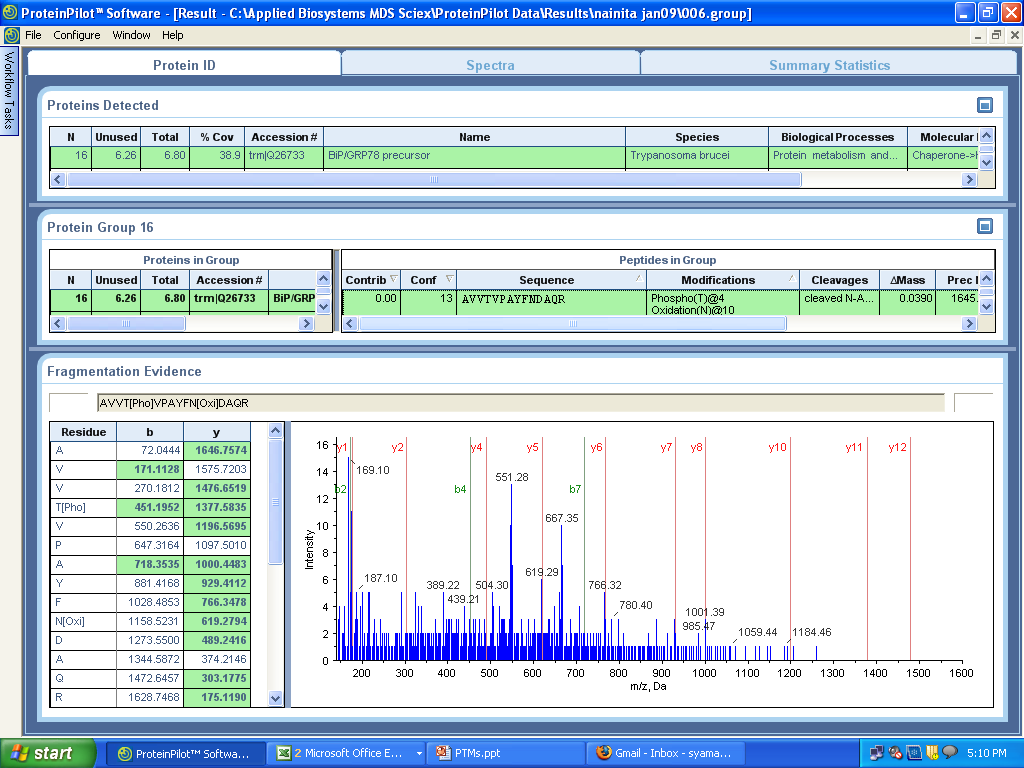


14.AVVT[Pho]VPAYFN[Oxi]DAQR: T – phosphorylation and N - oxidation:

Q26733: BiP/GRP78 precursor

13. ELGTVTDTAELQK: N terminal –acetylation: Q226840: Variable surface

glycoprotein


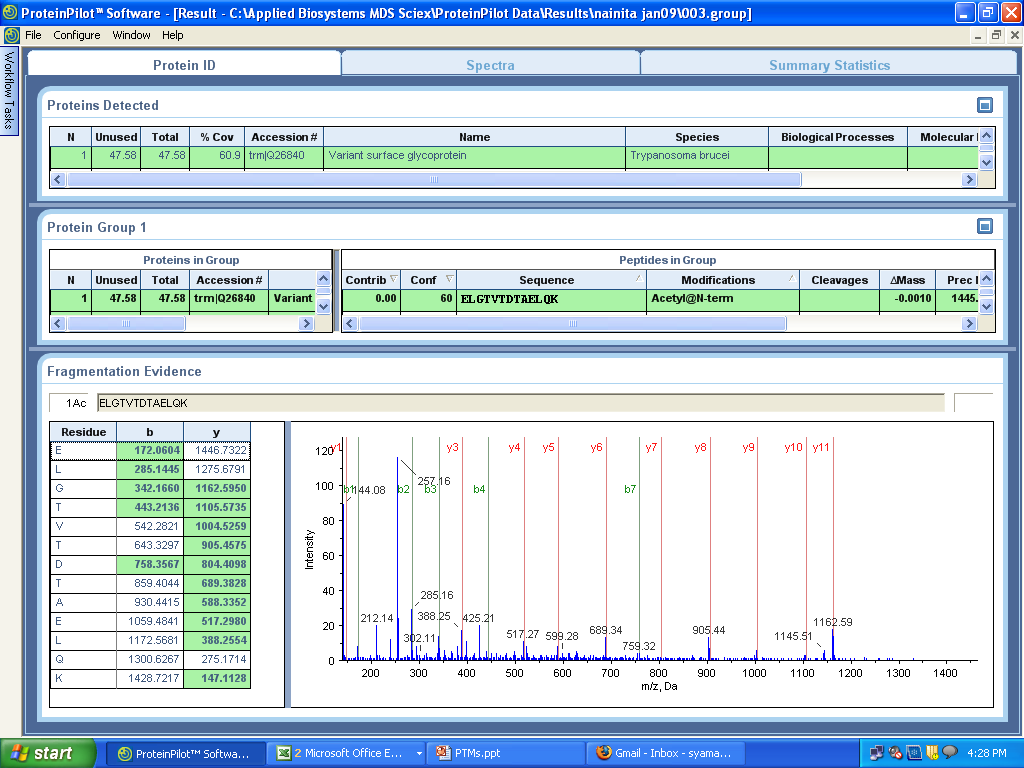

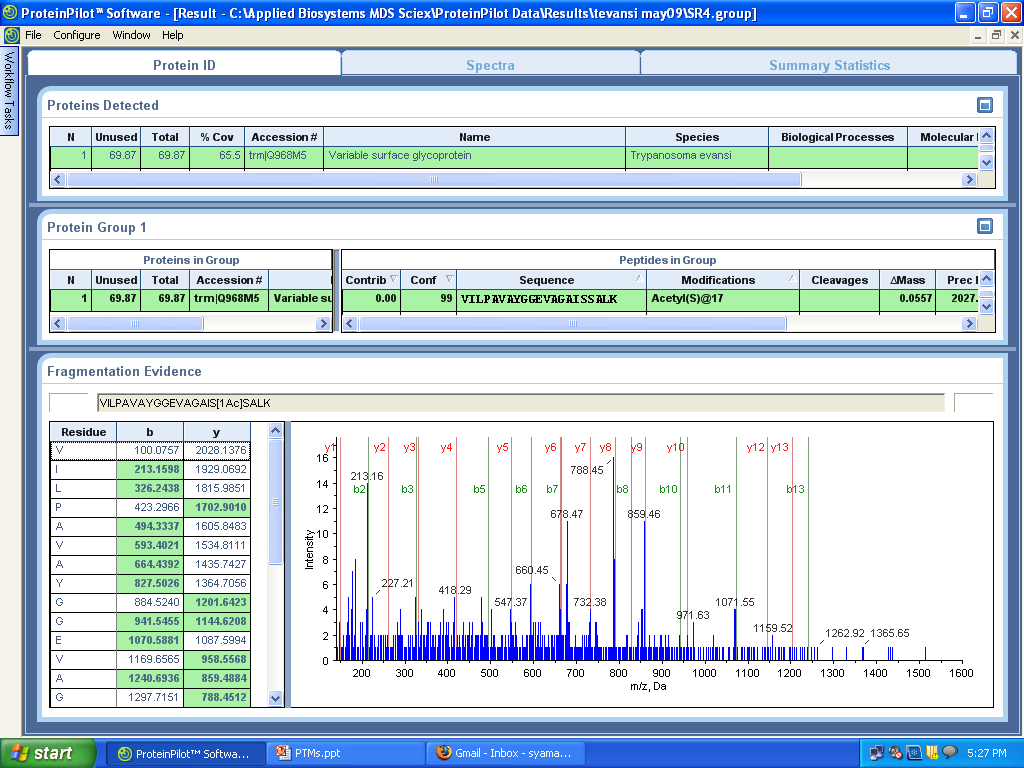


15. VILPAVAYGGEVAGAIS[Ac]SALK: S – acetylation: Q968M5:

Variable surface glycoprotein


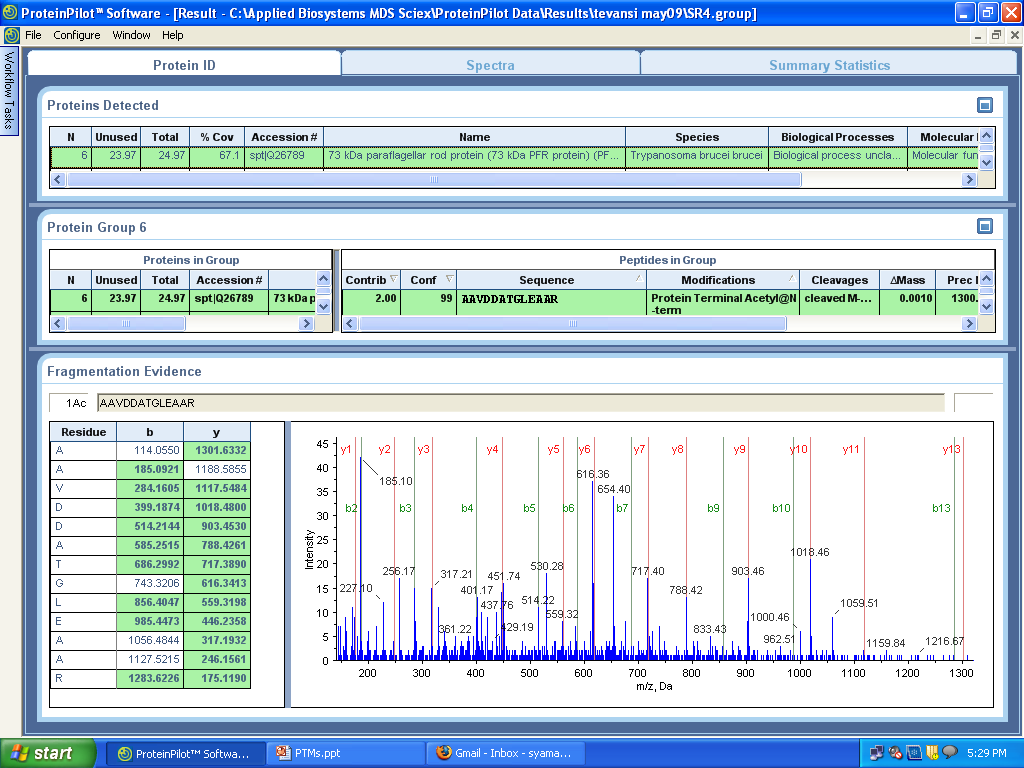


16. AAVDDATGLEAAR: N terminal –acetylation: Q26789:

73KDa paraflagellar rod protein


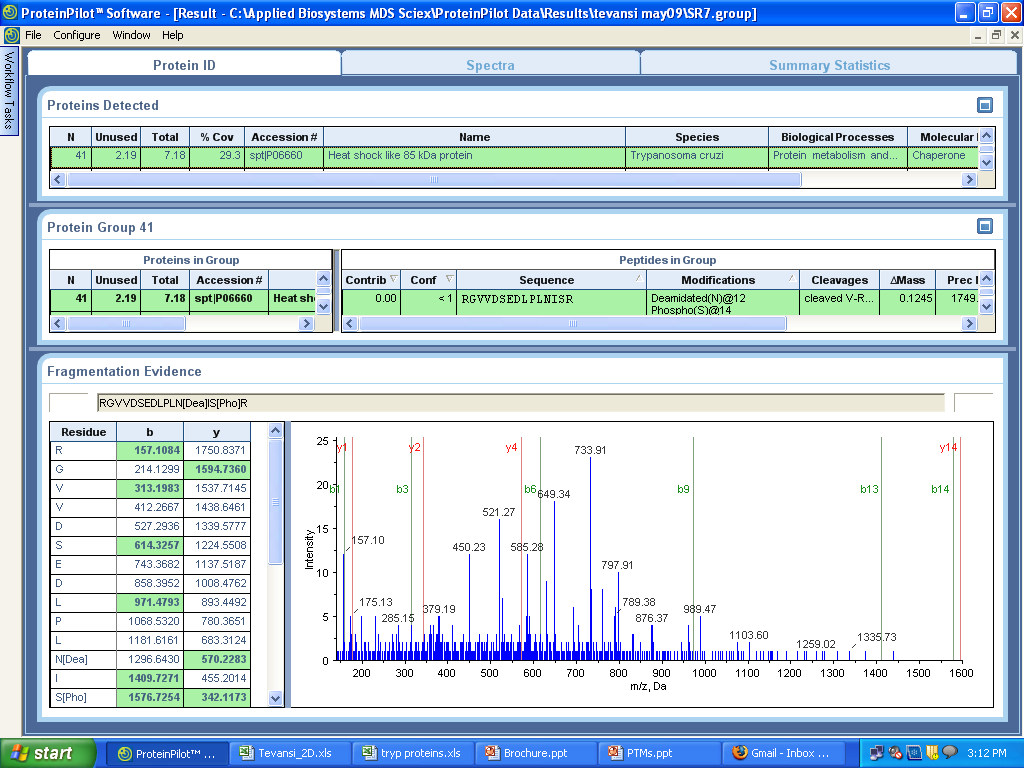


17. RGVVDSEDLPLNISR: S – phosphorylation: P06660: Heat shock like 85KDa protein
